# An Interdisciplinary Perspective on Improving Cancer Care in the State of Mississippi as an Example of Cancer Care Improvements in the Global South

**DOI:** 10.7759/cureus.76865

**Published:** 2025-01-03

**Authors:** Madison L Jones, Srinivasan Vijayakumar, Mary R Nittala, Claude D Brunson

**Affiliations:** 1 Medical Education, Mississippi State Medical Association, Ridgeland, USA; 2 Radiotherapy and Oncology, Kasturba Medical College, Manipal Academy of Higher Education, Manipal, IND; 3 Radiation Oncology, University of Chicago, University of Illinois Chicago, University of California, University of Mississippi Medical Center, Ridgeland, USA; 4 Cancer Care, Cancer Care Advisors and Consultants LLC, Ridgeland, USA; 5 Radiation Oncology, University of Mississippi Medical Center, Jackson, USA; 6 Medical Affairs, Mississippi State Medical Association, Ridgeland, USA; 7 Anesthesiology, University of Mississippi Medical Center, Jackson, USA

**Keywords:** cancer care, cancer disparities, deep south, global south, health inequities, health policy, mississippi, precision medicine, sdi, social determinants

## Abstract

Cancer disparities, a critical public health issue, particularly in states such as Mississippi, where socioeconomic factors significantly influence health outcomes, require our collective attention. This paper delves into the multifaceted nature of cancer disparities through a macro-level analysis of cancer data, specifically focusing on Mississippi as a microcosm of broader national and global trends. Two key indices, the Socio-Demographic Index (SDI) and the Social Deprivation Index (SDeI), provide valuable insights. The former offers a macro-level understanding of the socioeconomic factors that shape health and cancer outcomes. The latter quantifies disadvantages in small areas, identifying regions that need scientific, policy, and administrative support. The poor health care and cancer care (CC) outcomes in Mississippi are well documented and detailed here. However, SDI and SDeI data are not yet available in Mississippi. With biological, technological, and clinical research design advancements and other new innovative strategies emerging in the past decade in CC, a 'leapfrogging' of CC outcomes in Mississippi is within our reach. To achieve this goal, an interdisciplinary approach (IDA) addressing and solving the challenges faced in Mississippi is required. The IDA team must include disciplines that can determine SDI and SDeI for Mississippi and tie those findings to successfully apply new technological advances and innovations efficiently and cost-effectively by building infrastructure and developing implementation strategies. This can serve as a pilot demonstration project that will also help other similar regions within the United States, as well as the Global South.

## Introduction and background

Cancer is a pressing public health issue globally, with its impact felt disproportionately across different regions and populations. In the United States, the state of Mississippi presents a compelling case study of cancer care (CC) disparities and the complex interplay of factors influencing patient outcomes. Despite overall progress in cancer treatment in recent decades, certain populations have experienced little benefit from advances in CC, with some disparities [1,2] between populations of low and high socioeconomic status widening during this period, particularly for cancers where prevention and early screening could make the biggest difference [3]. This paper delves into the current state of health and CC in Mississippi, highlighting the urgent need to address the challenges faced by residents in accessing and receiving adequate health and CC screening, prevention, and treatment services in a timely manner, thus improving the outcomes and quality of life.

The healthcare status of Mississippians is a crucial lens through which we can understand the complexities of CC in the state. This paper aims to illuminate the multifaceted barriers contributing to disparities in cancer incidence, treatment, and survival rates. A central focus of this discussion is the pivotal role played by social determinants of health (SDOHs), such as socioeconomic status, education, and healthcare access, which play pivotal roles in shaping both cancer risk and quality of care [3].

Amidst the rapid advances in the evolution of oncology's CC management, new technological breakthroughs are offering promising new avenues for improving CC among disadvantaged populations. This study explores the potential of these advancements and how they might be leveraged to address the specific challenges faced in Mississippi and the greater south of the USA. Our primary objective is to identify strategies for ‘leapfrogging’ CC improvement in the state, instilling hope for a brighter future by taking an interdisciplinary approach.

By examining innovative approaches and formulating evidence-based recommendations, this paper seeks to contribute to the ongoing dialogue on reducing health disparities and improving cancer outcomes for all populations. Through a comprehensive analysis of Mississippi's CC landscape, we aim to provide a roadmap for closing the gap in care disparities, ensuring the credibility and effectiveness of our proposed strategies.

## Review

Healthcare status of Mississippians

The state of Mississippi, nestled in the Deep South of the United States, is filled with a rich culture and history shared by its residents. However, this cultural wealth stands in stark contrast to the urgent and long-standing healthcare challenges that have consistently placed Mississippi at the bottom of national health rankings. The current healthcare status of Mississippians is the result of a culmination of socioeconomic and systemic factors that greatly affect health outcomes, including CC outcomes. The following data are sourced from the Mississippi Health Disparities and Inequity Report, developed by the Mississippi State Department of Health (any verbatim reproduction, such as figures or tables, is done with permission and acknowledgment).

In 2010, researchers from the University of Mississippi Medical Center (UMMC) and Mississippi State University's (MSU) Social Science Research Center published a seminal report titled "What If We Were Equal: A Mississippi Health Assessment" [4]. This comprehensive and in-depth study delved into the sources of health disparities within the state, which include poverty, low median income, low education-attainment, and rural status [4].

According to the US Census, the state has one of the nation's highest poverty rates, with 20.3% of its population living below the poverty line in 2019. This economic hardship disproportionately affects Black Mississippians, who are more than twice as likely (31.6%) as their white counterparts (12.8%) to experience poverty. The state's median household income ($49,111) also ranks among the lowest in the country, with a significant racial gap evident: Black households earn a median income ($36,792) that is about half that of white households ($65,012). As Mississippi contends with one of the United State's lowest high school graduation rates, educational attainment further reveals troubling inequities. While Black adults have a slightly higher likelihood of not completing high school (17.4%) compared to White adults (14.2%), they are notably less likely to obtain a bachelor's degree (12.0% vs. 19.9%). This educational divide is mirrored in school performance, where all "F"-rated school districts are majority Black adults, while "A"-rated schools are predominantly White adults. Mississippi's rural status is compounding these challenges, with over half its population (51.2%) residing in rural areas - a proportion exceeded by only three other states. This geographic distribution contributes to an inequitable allocation of healthcare resources, as more than half of the state's doctors practice in just four urban centers, leaving many of Mississippi's 82 counties medically under-served and exacerbating the health vulnerabilities of rural residents.

**Figure 1:**
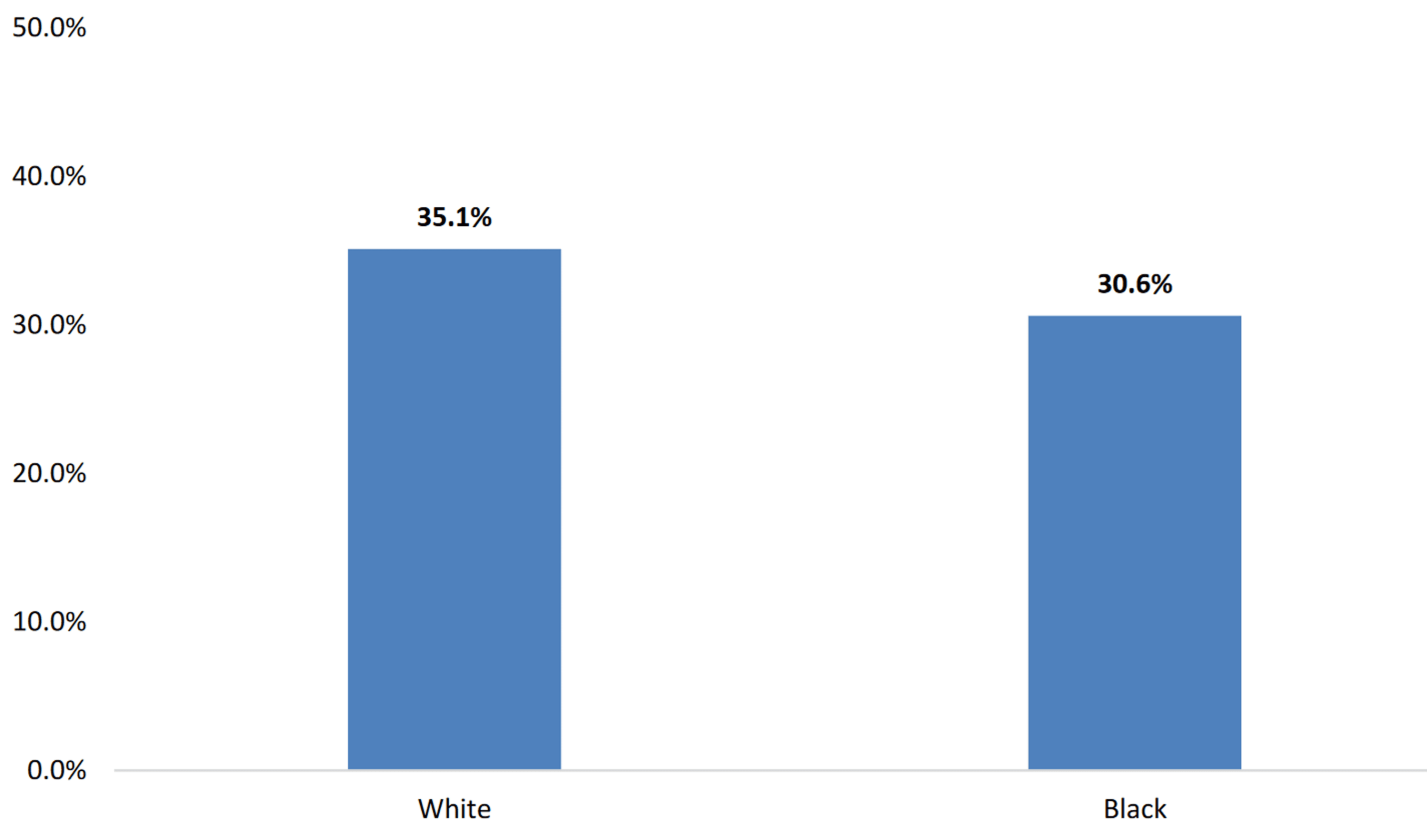
Overweight prevalence by race among Mississippi adults (2021). Note: The Y-axis is the percentage of the adult population in the state of Mississippi. This image is reproduced from [4], and permission was obtained from the publisher, Mississippi State Department of Health.

**Figure 2:**
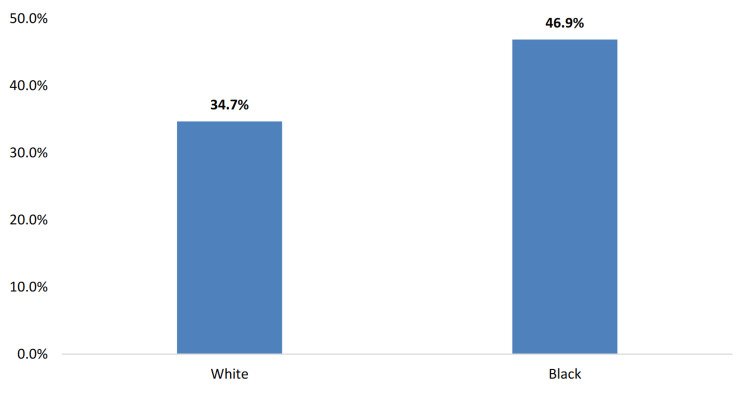
Obesity prevalence by race among Mississippi adults (2021). Note: The Y-axis is the percentage of the adult population in the state of Mississippi. This image is reproduced from [4], and permission was obtained from the publisher, Mississippi State Department of Health.

In connection to the differences in socioeconomic status across demographics, Mississippi exhibits concerning health disparities that underscore the complex interplay between social determinants and health outcomes. From Figure 1, initial data on overweight prevalence reveal a slightly higher rate among White Mississippians (35.1%) compared to Black Mississippians (30.6%). However, this pattern dramatically reverses when examining obesity, a more severe manifestation of weight-related health issues. Obesity prevalence is significantly higher among Black adults (46.9%) than among White adults (34.7%), a stark and urgent disparity that may reflect differences in access to nutritious food, safe spaces for physical activity, and quality healthcare (Figure 2).

**Figure 3:**
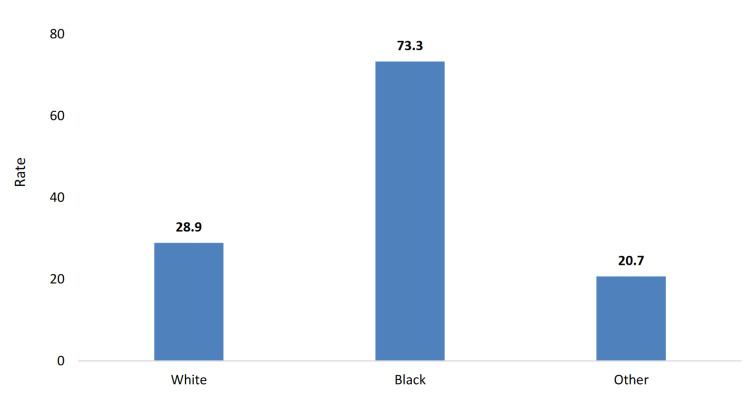
Age-adjusted diabetes mortality rate by race among Mississippi residents (2021). This image is reproduced from [4], and permission was obtained from the publisher, Mississippi State Department of Health.

The racial gap in age-adjusted diabetes mortality rates, as depicted in Figure 3, is a cause for alarm. Black Mississippians face a mortality rate of 73.3 deaths per 100,000 population, more than double the rate for White residents at 28.9 [4]. This stark contrast underscores the more significant barriers Black residents face in managing these conditions effectively, possibly due to limited access to specialized care, medications, and diabetes education programs. The progression from overweight status to obesity and ultimately to diabetes-related death highlights how initial health disparities can compound and result in severe outcomes disproportionately affecting minority communities within the state.

Multi-factorial causes that result in poor healthcare status and outcomes among Mississippians detailed above are not different from the factors that lead to poor CC outcomes in Mississippi.

CC outcome status of Mississippians and its relationship with income and education

**Figure 4:**
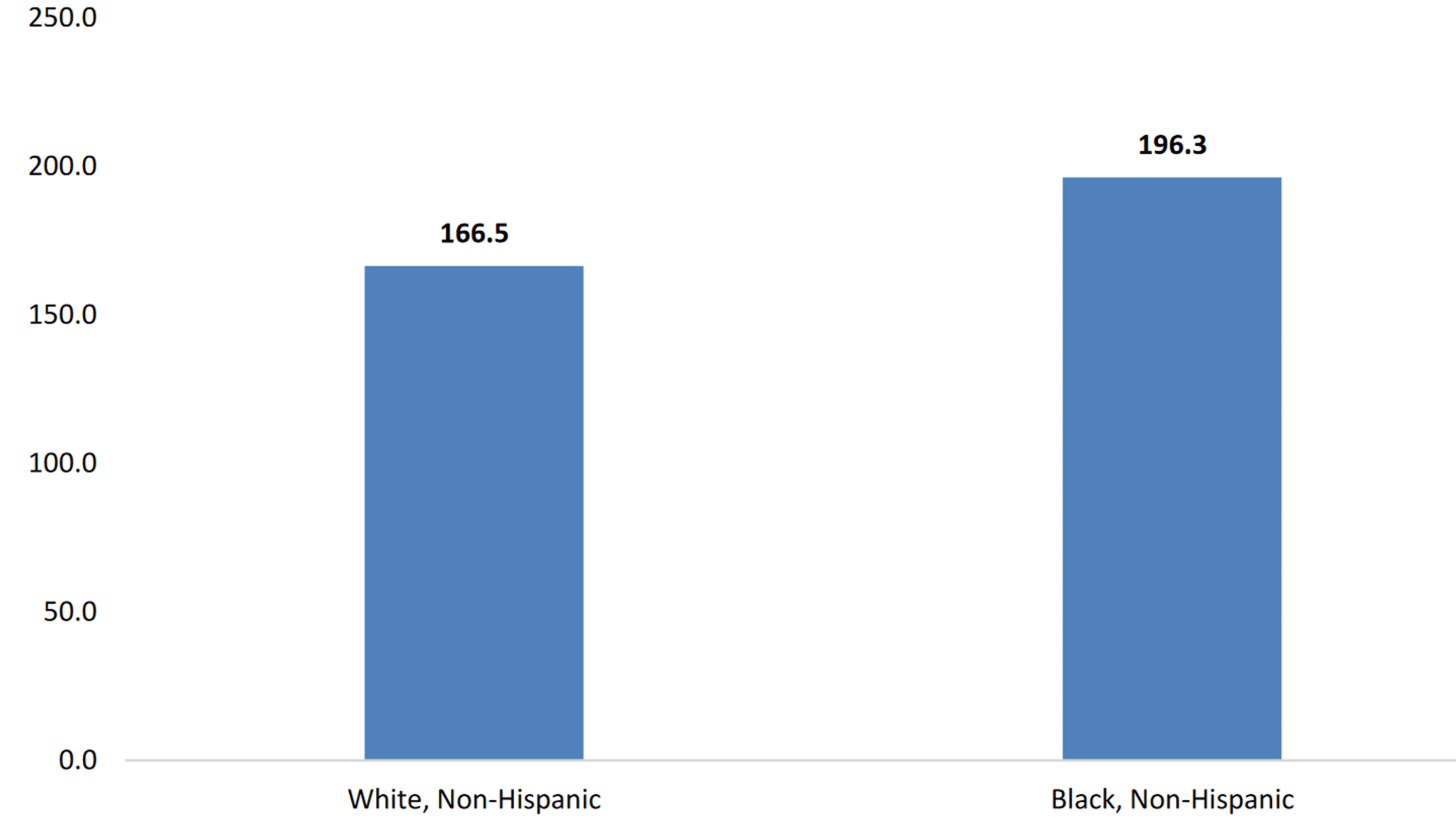
Age-adjusted cancer mortality rates by race among Mississippi residents (2020). Note: The Y-axis is the age-adjusted cancer mortality rate per 100,000 Mississippi population. The age-adjusted cancer mortality rate is 196.3 deaths among Black Mississippians and 166.5 deaths among White Mississippians, per 100,000 population. This image is reproduced from [4], and permission was obtained from the publisher, Mississippi State Department of Health.

**Figure 5:**
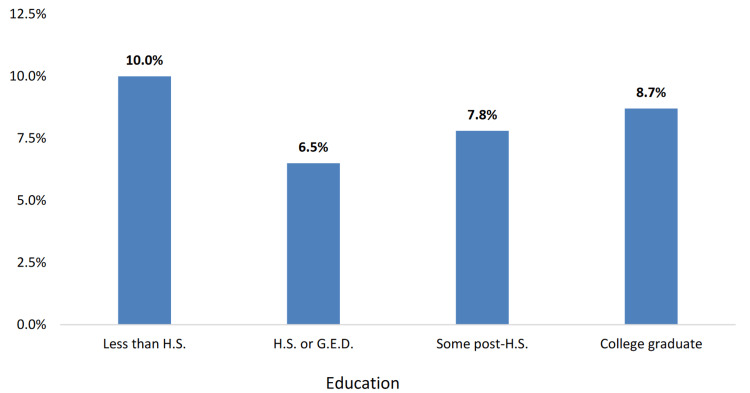
Cancer prevalence (excluding skin cancer) by education level among Mississippi adults. This image is reproduced from [4], and permission was obtained from the publisher, Mississippi State Department of Health. Note: There is a statistically significant educational disparity in the prevalence of having other types of cancer among adult population in Mississippi. Other types of cancer prevalence (10.0%), by education, is the highest among Mississippi adults with less than a high school degree.

**Figure 6:**
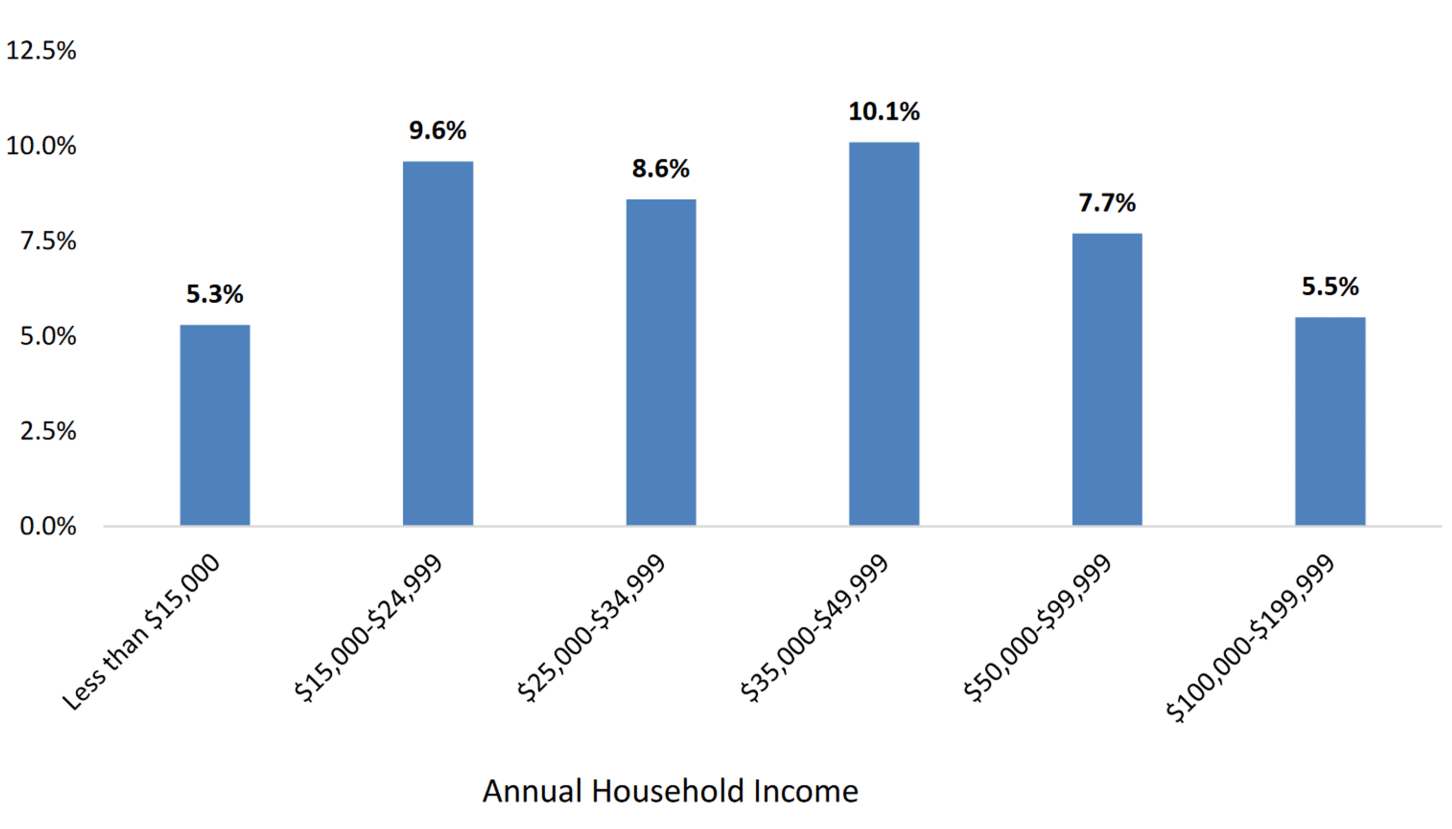
Cancer prevalence (excluding skin cancer) by annual household income among Mississippi adults (2021). This image is reproduced from [4], and permission was obtained from the publisher, Mississippi State Department of Health. Note: There is a statistically significant income disparity in the prevalence of having other types of cancer. Other types of cancer prevalence (10.1%), by annual household income, is the highest among Mississippi adults earning between $35,000 and $49,999. The Y-axis is the percentage of adults in the state of Mississippi, within each income range.

**Figure 7:**
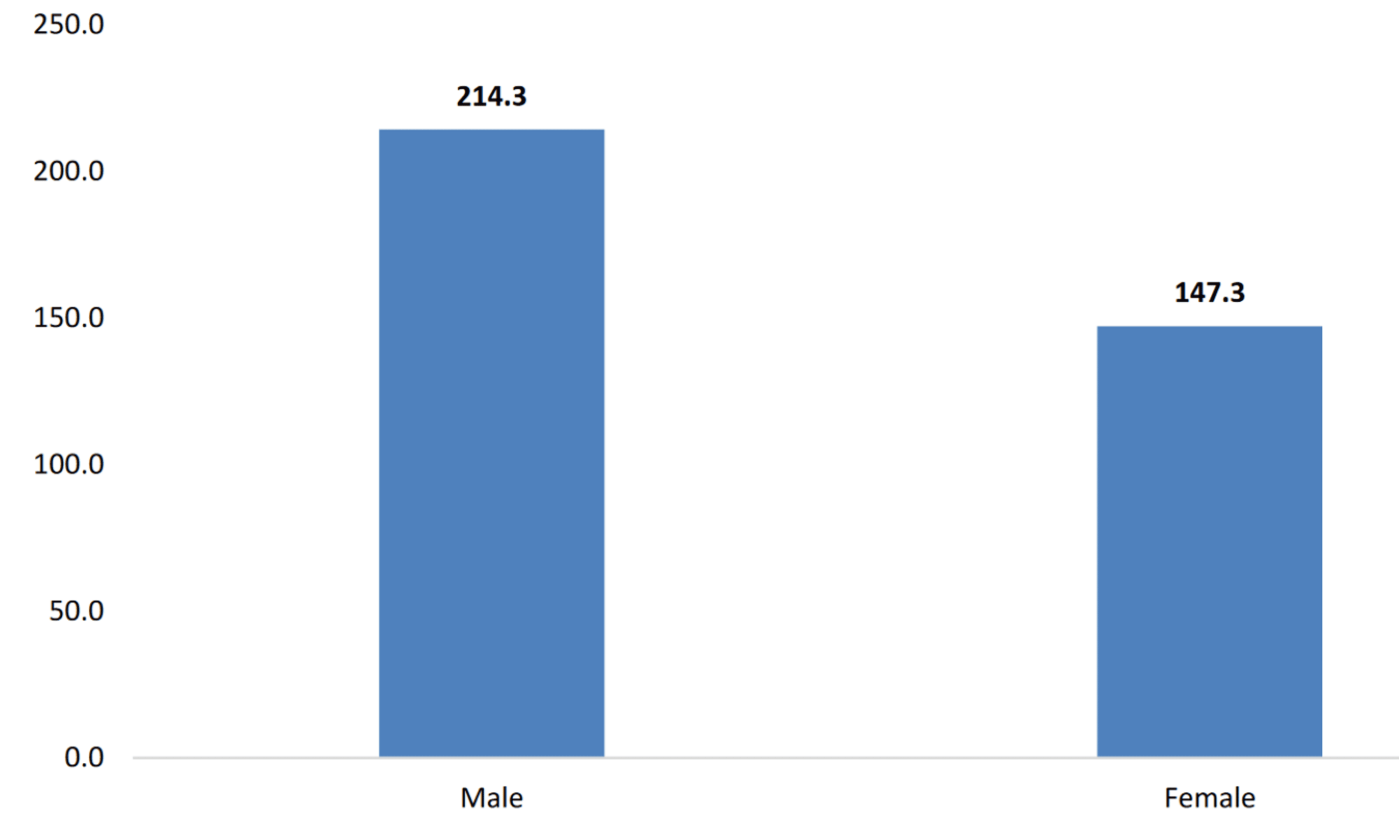
Age-adjusted cancer mortality rates by gender among Mississippi residents (2020). This image is reproduced from [4], and permission was obtained from the publisher, Mississippi State Department of Health. Note: The age-adjusted cancer mortality rate is 214.3 deaths among male Mississippians and 147.3 deaths among female Mississippians, per 100,000 population. The Y-axis is the percentage of adult male/female with age-adjusted cancer mortality rate, per 100,000 population.

The socioeconomic factors affecting Mississippi’s diverse population contribute to significant disparities in cancer incidence, mortality, and access to quality healthcare. As illustrated in Figure 4, the age-adjusted cancer mortality rate is higher among Black Mississippians (196.3 deaths per 100,000 population) compared to White Mississippians (166.5 deaths per 100,000 population). Educational disparities also exist, with the prevalence of other types of cancer being highest (10.0%) among Mississippi adults with less than a high school degree (Figure 5). Income disparities are evident as well, with the prevalence of other types of cancer being the highest (10.1%) among Mississippi adults earning between $35,000 and $49,999 annually (Figure 6). Furthermore, in Figure 7, gender differences are observed in cancer mortality rates, with male Mississippians having a higher age-adjusted mortality rate (214.3 deaths per 100,000 population) compared to female Mississippians (147.3 deaths per 100,000 population).

Mississippi faces several significant obstacles to providing its residents with comprehensive and equitable CC. One of the primary challenges is the high prevalence of poverty and low levels of education in the state, which are known to be associated with increased cancer risk factors, delayed diagnosis, and poorer treatment outcomes [3]. A recent nationwide study examining Black and White adults in the United States revealed that those who had not completed high school faced a markedly higher risk of dying from cancer compared to high school graduates [5]. The study also found that social determinants of health (SDOHs) partially explained the link between an individual's education level and cancer mortality risk. In contrast, health behaviors did not appear to be a significant factor [3]. These insights suggest that tackling SDOHs, such as improving access to health insurance and bolstering public health systems, could reduce cancer deaths among less-educated populations [5].

The disparity in cancer mortality rates based on education level has been widening over time [6]. An extensive analysis of US death records, encompassing 8.2 million individuals, revealed that between 1989 and 2018, age-adjusted cancer mortality rates climbed among those with less than 12 years of schooling while simultaneously decreasing for those with 12 or more years of education [6]. This widening gap suggests that the benefits of advancements in cancer prevention, detection, and treatment are not reaching all segments of society equally. For states such as Mississippi, where educational disparities are particularly pronounced, these findings underscore the pressing need for targeted strategies to enhance CC accessibility and quality across all academic backgrounds.

Additionally, Mississippi's rural landscape and limited public transportation options make it difficult for many residents, particularly those in underserved communities, to access specialized CC facilities and services. A study assessing the perspectives of rural cancer patients and caregivers found that many patients struggle with insufficient financial resources to cover long-distance travel costs [7]. In contrast, rural areas’ need for robust public transportation further complicates access. The extensive time required for these journeys poses additional difficulties, as patients must balance treatment needs with work and family obligations [7]. Moreover, the resource-intensive nature of these trips, often requiring expenses for lodging and meals, adds another layer of financial strain. These distance-related challenges can lead to delayed or inconsistent care, potentially compromising treatment outcomes and quality of life for rural cancer patients [7].

The state also faces a shortage of healthcare professionals, including oncologists and primary care physicians, which further exacerbates the problem of access to quality CC. According to a study forecasting physician workforce shortage in the United States, Mississippi is the only state projected to score an “F” grade for physician availability [8]. The state has 118 active physicians per 100,000 population, 42% below the national mean of 203 [8]. To achieve parity with the national benchmark by 2030, Mississippi will require an additional 3,709 physicians, representing a 51% increase from the state's 2017 physician workforce of 3,528 [8]. Despite these challenges, Mississippi’s medical education institutions, encompassing both MD and DO programs, have exhibited the most significant percentage increase in enrollment nationwide [8]. This expansion has resulted in a growth rate surpassing 130%, indicating a concerted effort to bolster the future physician workforce within the state [8]. Mississippi's healthcare infrastructure, including hospitals and cancer treatment centers, may lack the advanced technologies and resources necessary to provide state-of-the-art CC, especially in rural areas. These obstacles, combined with the complex interplay of socioeconomic, racial, and geographic factors, contribute to the persistent cancer disparities observed in Mississippi, emphasizing the need for targeted interventions and policies to address these challenges and ensure equitable access to high-quality CC for all Mississippians.

Importance of SDI and SDeI in healthcare and CC outcomes

**Figure 8:**
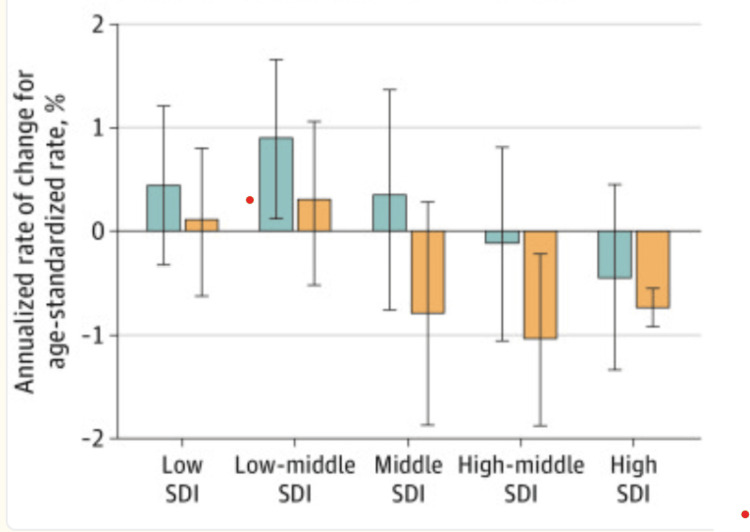
Differences between 2010 and 2019 in terms of the annualized rate of change for the age-standardized rate. The largest increasing annualized rates of change in the absolute numbers of cases and deaths occurred in the low-middle SDI quintile and then the low SDI quintile. Abbreviations: SDI = Social Demographic Index; black bars = 95% uncertainty intervals; green bars = incidence; yellow bars = mortality This image is reproduced from Global Burden of Disease 2019 Cancer Collaboration including the caption and legend and is available via Creative Commons Attribution License. Note: Global estimates for total cancers, except nonmelanoma skin cancer, stratified by SDI quintile.

**Figure 9:**
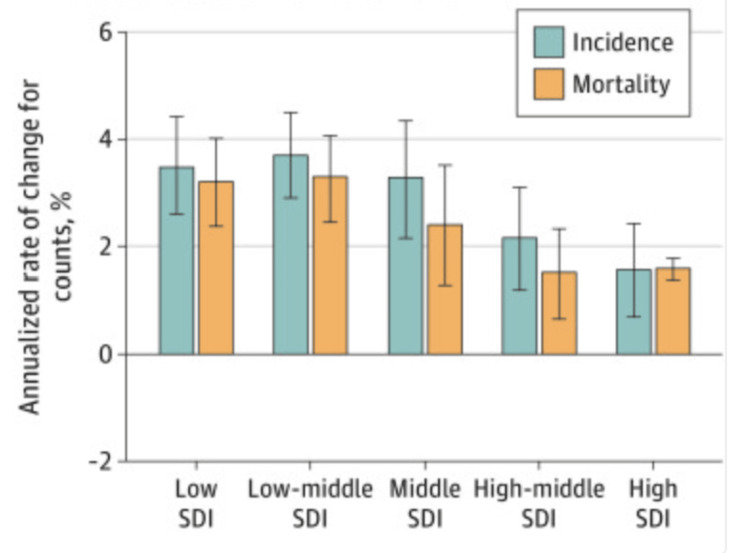
Changes between 2010 and 2019 in terms of counts. This image is reproduced from Global Burden of Disease 2019 Cancer Collaboration including the caption and legend and is available via Creative Commons Attribution License. Note: Total cancer incidence and mortality age-standardized rates and absolute counts in 2019 and annualized rate of change for incidence and mortality in age-standardized rates and absolute counts from 2010 to 2019 by sociodemographic index (SDI) quintile Global estimates for total cancers, except nonmelanoma skin cancer, stratified by SDI quintile. Annualized rate of change from 2010 to 2019 represents the mean percentage change per year during this period. Black bars represent 95% uncertainty intervals.

Understanding and addressing the significant cancer disparities in Mississippi is a pressing issue that requires a macro-level analysis, with the socio-demographic index (SDI) playing a crucial role. Developed by researchers of the Global Burden of Disease Study (2019), SDI is a powerful indicator of a country’s health outcomes, strongly correlated with its development status [1]. This index, which combines data on the economy, education, and fertility rates, provides a comprehensive representation of social and economic development [1]. Specifically, the SDI is a composite indicator of income per capita, mean years of education, and fertility rate for those younger than 25 [1]. A lower score on the index’s scale indicates a minimal level of development. Conversely, a higher SDI value is indicative of a more advanced level of development [1]. These values are closely linked to life expectancy. Countries are categorized into quintiles based on their SDI values. The cancer burden has risen in all SDI quintiles since 2010 [1]. However, the largest percentage increases were observed in the low and low-middle quintiles (Tables 1-2, Figures 8-9) [1].

**Table 1:** Global incidence and mortality from total cancers and 29 cancer groups (2019). This table is reproduced from Global Burden of Disease 2019 Cancer Collaboration [1] and is available via Creative Commons Attribution License. Abbreviations: ASIR, age-standardized incidence rate; ASMR, age-standardized mortality rate; NA, not applicable; NMSC, nonmelanoma skin cancer; UI, uncertainty interval. ^a^Rows are ordered by decreasing number of total deaths. Cancer groups are defined based on International Classification of Diseases, Ninth Revision (ICD-9) and International Classification of Diseases and Related Health Problems, Tenth Revision (ICD-10) codes and include all codes pertaining to malignant neoplasms (ICD-9 codes 140-208 and ICD-10 codes C00-C96) except for Kaposi sarcoma (C46; ^e^Appendix in the supplement of the reference [1]).

Cancer type^a^	Deaths, thousands (95% UI)					ASMR per 100 000 (95% UI)					Incident cases, thousands (95% UI)					ASIR per 100 000 (95% UI)				
	Total	Male		Female		Total	Male		Female		Total	Male		Female		Total	Male		Female	
Total	10 000 (9360-10 600)		5690 (5250-6100)		4340 (3970-4660)	124.7 (116.4-132.0)		156.1 (143.9-167.2)		99.9 (91.5-107.3)	23 600 (22 200-24 900)		12 900 (12 100-13 800)		10 600 (9920-11 400)	290.5 (274.0-307.1)		348.7 (327.3-370.8)		246.1 (229.8-263.1)
Excluding NMSC	9970 (9310-10 500)		5650 (5220-6 070)		4310 (3950-4 640)	123.9 (115.7-131.2)		155.1 (142.9-166.1)		99.4 (91.0-106.8)	17 200 (15 900-18 500)		9260 (8470-10 000)		7960 (7280-8610)	211.4 (195.4-226.8)		245.9 (225.3-266.5)		185.0 (169.4-200.2)
Tracheal, bronchus, and lung	2040 (1880-2190)		1390 (1260-1510)		657 (590-719)	25.2 (23.2-27.0)		37.4 (34.1-40.7)		15.0 (13.5-16.4)	2260 (2070-2450)		1520 (1370-1680)		737 (658-814)	27.7 (25.3-30.0)		40.4 (36.5-44.4)		16.8 (15.0-18.6)
Colon and rectum	1090 (1000-1150)		594 (551-638)		492 (438-532)	13.7 (12.6-14.5)		16.6 (15.4-17.9)		11.2 (10.0-12.2)	2170 (2000-2 340)		1240 (1130-1360)		926 (832-1 010)	26.7 (24.6-28.9)		33.1 (30.2-36.2)		21.2 (19.0-23.2)
Stomach	957 (871-1030)		612 (544-678)		346 (308-382)	11.9 (10.8-12.8)		16.6 (14.8-18.3)		7.9 (7.1-8.8)	1270 (1150-1400)		847 (748-963)		423 (377-467)	15.6 (14.1-17.2)		22.4 (19.8-25.3)		9.7 (8.7-10.7)
Breast	701 (647-752)		12.1 (10.7-13.3)		689 (635-740)	8.6 (7.9-9.2)		0.3 (0.3-0.4)		15.9 (14.7-17.1)	2000 (1830-2170)		25.1 (22.2-27.8)		1980 (1810-2150)	24.2 (22.1-26.2)		0.7(0.6-0.7)		45.9 (41.9-49.8)
Pancreatic	531 (492-567)		278 (258-299)		253 (226-274)	6.6 (6.1-7.1)		7.5 (7.0-8.1)		5.8 (5.1-6.2)	530 (486-574)		280 (256-303)		250 (224-275)	6.6 (6.0-7.1)		7.5 (6.8-8.1)		5.7 (5.1-6.3)
Esophageal	498 (438-551)		366 (315-415)		133 (110-150)	6.1 (5.4-6.8)		9.7 (8.3-11.0)		3.0 (2.5-3.4)	535 (467-595)		389 (336-444)		146 (120-165)	6.5 (5.7-7.2)		10.1 (8.7-11.6)		3.3 (2.7-3.8)
Prostate	487 (420-594)		487 (420-594)		NA	6.3 (5.4-7.7)		15.3 (13.0-18.6)		NA	1410 (1230-1830)		1410 (1230-1830)		NA	17.4 (15.1-22.5)		38.6 (33.6-49.8)		NA
Liver	485 (444-526)		334 (300-368)		151 (134-167)	5.9 (5.4-6.4)		8.7 (7.9-9.6)		3.5 (3.1-3.8)	534 (487-589)		376 (335-422)		158 (140-176)	6.5 (5.9-7.2)		9.7 (8.7-10.8)		3.6 (3.2-4.0)
Other malignant neoplasms	408 (355-444)		220 (180-249)		188 (169-204)	5.1 (4.5-5.6)		5.9 (4.8-6.7)		4.5 (4.0-4.8)	831 (741-906)		451 (381-504)		381 (347-415)	10.4 (9.3-11.4)		11.9 (10.0-13.3)		9.2 (8.4-10.1)
Leukemia	335 (307-360)		188 (165-208)		146 (132-158)	4.3 (3.9-4.6)		5.2 (4.6-5.7)		3.5 (3.2-3.8)	644 (587-700)		351 (308-390)		293 (263-322)	8.2 (7.5-8.9)		9.4 (8.3-10.5)		7.2 (6.5-8.0)
Cervical	280 (239-314)		NA		280 (239-314)	3.4 (2.9-3.8)		NA		6.5 (5.5-7.3)	566 (482-636)		NA		566 (482-636)	6.8 (5.8-7.7)		NA		13.4 (11.4-15.0)
Non-Hodgkin lymphoma	255 (238-270)		146 (136-155)		109 (98.9-117)	3.2 (3.0-3.4)		4.0 (3.7-4.2)		2.5 (2.3-2.7)	457 (417-499)		266 (241-291)		191 (169-211)	5.7 (5.2-6.3)		7.2 (6.5-7.9)		4.5 (4.0-4.9)
Brain and central nervous system	246 (186-271)		139 (99.6-157)		108 (76.4-122)	3.0 (2.3-3.4)		3.6 (2.6-4.1)		2.6 (1.8-2.9)	348 (262-389)		187 (135-215)		161 (114-184)	4.3 (3.3-4.9)		4.8 (3.5-5.6)		3.9 (2.8-4.5)
Bladder	229 (211-243)		169 (157-181)		59.5 (52.3-64.6)	2.9 (2.7-3.1)		5.1 (4.7-5.4)		1.4 (1.2-1.5)	524 (476-569)		408 (371-444)		116 (104-128)	6.5 (5.9-7.1)		11.3 (10.2-12.3)		2.7 (2.4-2.9)
Lip and oral cavity	199 (182-218)		132 (118-145)		67.8 (60.8-75.7)	2.4 (2.2-2.7)		3.4 (3.1-3.8)		1.6 (1.4-1.7)	373 (341-404)		243 (219-268)		130 (117-143)	4.5 (4.1-4.9)		6.2 (5.6-6.8)		3.0 (2.7-3.3)
Ovarian	198 (175-218)		NA		198 (175-218)	2.4 (2.1-2.7)		NA		4.6 (4.0-5.0)	294 (261-330)		NA		294 (261-330)	3.9 (3.2-4.0)		NA		6.9 (6.1-7.7)
Gallbladder and biliary tract	172 (145-189)		73.0 (59.5-80.4)		99.5 (81.7-114.0)	2.2 (1.8-2.4)		2.1 (1.7-2.3)		2.3 (1.9-2.6)	199 (167-220)		86.4 (69.4-95.9)		113 (91.6-130)	2.5 (2.1-2.7)		2.4 (1.9-2.7)		2.6 (2.1-3.0)
Kidney	166 (155-176)		109 (101-116)		57.7 (52.2-61.9)	2.1 (1.9-2.2)		3.0 (2.8-3.2)		1.3 (1.2-1.4)	372 (345-402)		241 (221-262)		131 (120-142)	4.6 (4.2-4.9)		6.2 (5.7-6.8)		3.1 (2.8-3.3)
Larynx	123 (115-133)		106 (97.8-115)		17.8 (16.2-19.7)	1.5 (1.4-1.6)		2.7 (2.5-3.0)		0.4 (0.4-0.5)	209 (194-225)		181 (166-196)		28.5 (26.1-31.3)	2.5 (2.3-2.7)		4.6 (4.2-5.0)		0.7 (0.6-0.7)
Other pharynx	114 (103-126)		88.0 (78.0-98.7)		26.2 (22.5-30.5)	1.4 (1.2-1.5)		2.2 (2.0-2.5)		0.6 (0.5-0.7)	167 (153-180)		129 (116-142)		37.6 (33.1-42.3)	2.0 (1.8-2.2)		3.2 (2.9-3.5)		0.9 (0.8-1.0)
Multiple myeloma	113 (99.5-122)		60.4 (50.7-67.1)		53.0 (45.1-58.3)	1.4 (1.2-1.5)		1.7 (1.4-1.8)		1.2 (1.0-1.3)	156 (137-173)		84.5 (70.9-94.9)		71.2 (60.3-80.1)	1.9 (1.7-2.1)		2.3 (1.9-2.6)		1.6 (1.4-1.8)
Uterine	91.6 (82.4-101.5)		NA		91.6 (82.4-101.5)	1.1 (1.0-1.3)		NA		2.1 (1.9-2.3)	435 (397-480)		NA		435 (397-480)	5.2 (4.8-5.7)		NA		10.0 (9.1-11.0)
Nasopharynx	71.6 (65.4-77.6)		51.2 (46.0-57.0)		20.4 (18.2-22.8)	0.9 (0.8-0.9)		1.3 (1.2-1.4)		0.5 (0.4-0.5)	177 (156-200)		127 (108-149)		49.2 (42.6-57.0)	2.1 (1.9-2.4)		3.1 (2.7-3.7)		1.2 (1.0-1.3)
Malignant skin melanoma	62.8 (46.3-71.0)		35.4 (22.0-42.7)		27.4 (19.0-31.9)	0.8 (0.6-0.9)		1.0 (0.6-1.2)		0.6 (0.4-0.7)	290 (214-342)		153 (89.8-193)		137 (92.7-167)	3.6 (2.6-4.2)		4.0 (2.3-5.1)		3.2 (2.2-3.9)
Nonmelanoma skin	56.1 (50.4-59.8)		33.2 (30.3-35.6)		22.8 (19.3-25.2)	0.7 (0.7-0.8)		1.0 (0.9-1.1)		0.5 (0.4-0.6)	6350 (5810-6950)		3680 (3350-4060)		2670 (2430-2910)	79.1 (72.3-86.6)		102.8 (93.9-112.9)		61.1 (55.8-66.7)
Thyroid	45.6 (41.3-48.8)		18.6 (16.8-20.2)		26.9 (23.7-29.3)	0.6 (0.5-0.6)		0.5 (0.5-0.6)		0.6 (0.5-0.7)	234 (212-253)		76.0 (68.2-82.9)		158 (140-173)	2.8 (2.6-3.1)		1.9 (1.7-2.1)		3.7 (3.3-4.1)
Mesothelioma	29.3 (26.7-31.0)		21.2 (20.0-22.5)		8.03 (5.88-8.92)	0.4 (0.3-0.4)		0.6 (0.6-0.6)		0.2 (0.1-0.2)	34.5 (31.2-37.8)		25.2 (22.9-27.6)		9.34 (6.84-10.7)	0.4 (0.4-0.5)		0.7 (0.6-0.8)		0.2 (0.2-0.2)
Hodgkin lymphoma	27.6 (23.7-31.8)		17.2 (13.9-21.0)		10.4 (8.23-12.6)	0.3 (0.3-0.4)		0.4 (0.4-0.5)		0.3 (0.2-0.3)	87.5 (77.9-101.4)		51.3 (43.6-58.7)		36.2 (30.2-46.1)	1.1 (1.0-1.3)		1.3 (1.1-1.5)		0.9 (0.7-1.1)
Testicular	10.8 (9.96-11.9)		10.8 (9.96-11.9)		NA	0.1 (0.1-0.2)		0.3 (0.3-0.3)		NA	109.3 (93.4-129.5)		109.3 (93.4-129.5)		NA	1.4 (1.2-1.7)		2.8 (2.4-3.3)		NA

**Table 2:** Global cancer estimates and rankings among disease categories by the sociodemographic index (2019). This table is reproduced from Global Burden of Disease 2019 Cancer Collaboration [1] and is available via Creative Commons Attribution License. Abbreviations: DALYs, disability-adjusted life years; SDI, Sociodemographic Index; UI, uncertainty interval; YLDs, years lived with disability; YLLs, years of life lost. ^a^Total numbers and rankings exclude nonmelanoma skin cancer. All estimates refer to estimates in 2019. Rank refers to the relative ranking of the total cancer estimate for a given measure (e.g., DALYs) and SDI quintile (e.g., high SDI) compared among the 22 level two categories of diseases and injuries in the Global Burden of Disease Study (2019). The implications when compared with 2010 statistics are expanded on Figures 8-9.

Location^a^	DALYs (disability-adjusted life years)		Deaths		YLLs (years of life lost)		Incident cases		YLDs (years lived with disability)	
	No. millions (95% UI)	Cancer rank	No. millions (95% UI)	Cancer rank	No. millions (95% UI)	Cancer rank	No. millions (95% UI)	Cancer rank	No. millions (95% UI)	Cancer rank
Global	249.0 (233.6-263.2)	2	9.97 (9.31-10.5)	2	241.3 (226.5-255.3)	2	17.2 (15.9-18.5)	21	7.72 (5.68-9.96)	20
SDI										
Low	18.0 (15.9-20.2)	10	0.54 (0.48-0.60)	5	17.7 (15.7-19.8)	9	0.68 (0.60-0.76)	21	0.26 (0.18-0.34)	22
Low-middle	40.2 (36.8-43.7)	4	1.37 (1.26-1.49)	2	39.5 (36.1-43.0)	4	1.81 (1.67-1.96)	21	0.70 (0.52-0.92)	22
Middle	76.3 (69.7-83.2)	2	2.88 (2.62-3.15)	2	74.5 (68.0-81.4)	2	4.47 (4.05-4.89)	21	1.85 (1.36-2.44)	20
High-middle	63.5 (58.6-68.2)	2	2.65 (2.42-2.85)	2	61.4 (56.6-66.0)	2	4.69 (4.29-5.09)	21	2.11 (1.54-2.75)	16
High	50.9 (48.1-52.9)	1	2.53 (2.31-2.64)	2	48.1 (45.5-49.7)	1	5.56 (5.02-6.09)	19	2.79 (2.03-3.61)	12

This trend is concerning, as it indicates that the burden of cancer is disproportionately increasing in under-resourced countries with existing disparities in healthcare access and coverage. Moreover, lower SDI quintiles lack sufficient access to cancer prevention services, timely diagnosis, and comprehensive treatment, exacerbating the problem [1]. The categorization of countries into SDI quintiles should not be interpreted as suggesting uniform cancer prevention, diagnostic, or treatment capabilities within each quintile; each country has unique strengths and requirements that warrant individual consideration [1].

Survival rates in countries worldwide have improved significantly since 1950, with thousands of deaths averted due to the progress made. This improvement, while significant, also highlights the potential for further progress. However, the level of progress made is unequal for different nations, different geographics within each nation, and different ethnic groups. Similarly to the United States, cancer mortality is heavily influenced by the rates among the lower educational groups, even in countries we consider to be advanced [1].

Mississippi is a microcosm of the above facts and can be used as an example. The Social Deprivation Index (SDeI) was developed by Butler et al. in 2012 using the 2005-2009 American Community Survey (ACS) data for Primary Care Service Areas (PCSA) [9] (the details of the narrative on SDeI is sourced from the Robert Graham Center - Policy Studies in Family Medicine & Primary Care, with no in verbatim reproductions) [9]. The SDeI was later updated by researchers at the Graham Center with newer data and expanded to include counties, census tracts, zip code tabulation areas (ZCTA), and primary care services areas. To construct the PCSA-level SDeI, all of the ACS population measures were extracted at the census tract level and then summarized at the PCSA level. The SDeI measures for the county, census tract, and ZCTA levels were calculated using population data directly from the ACS. These SDeI scores are now accessible for every county, census tract, ZCTA, and PCSA in the dataset [2].

The SDI is used to quantify levels of disadvantage in small areas to identify areas that need additional healthcare resources. This SDeI is a composite measure of seven demographic characteristics: the percentage living in poverty, the percentage with less than 12 years of education, the percentage of single-parent households, the percentage living in rented housing units, the percentage living in overcrowded housing units, percentage of households without a car, and percentage of non-employed adults under 65 years of age. Each factor was chosen for its significant impact on health outcomes and healthcare resource needs [2]. Understanding both indices and the factors that determine the data allows for a deeper comprehension of the disparities in Mississippi.

While the SDeI is a powerful tool for identifying areas needing additional healthcare resources, it has its limitations. For example, it may not fully capture the complexity of social deprivation in all communities, and it may not account for other important factors that influence health outcomes. Understanding these limitations is crucial for interpreting the SDeI and using it effectively in healthcare resource allocation. The SDeI was developed using factor analysis on 14 candidate measures from the ACS. Factor analysis identifies underlying factors explaining relationships among variables. The measures were standardized to centiles for easier interpretation. Robert Graham Center researchers then refined the model by including only measures with factor loadings above 0.60, excluding the percentage of black and high-needs age groups [2]. The final SDeI was based on weighted factor loading scores. Models were compared using non-employed vs. unemployed measures, and slightly higher factor loading was found for the non-employed model. This process was replicated across different periods and geographic levels to ensure consistency and reliability of the SDeI across various contexts [2].

Each component of the SDeI is calculated using specific formulas to ensure an accurate measurement of social deprivation. The percent of the population living in poverty is calculated as the population below 0.99 of the federal poverty level divided by the total population [2]. Educational attainment is measured by the population aged 25 years or more with less than 12 years of education divided by the total population [2]. The non-employment rate for the working-age population (16-64 years) is determined by adding the population not in the labor force and the unemployed between 16 and 64 years, divided by the sum of the civilian population and those not in the labor force between 16 and 64 years [2]. Housing conditions are assessed through two metrics: the percentage of renter-occupied housing units (calculated as renter-occupied divided by the sum of owner-occupied and renter-occupied units) and overcrowded housing (measured by tenure by occupants per room minus the sum of owner-occupied and renter-occupied, divided by total occupied housing units) [2]. The proportion of single-parent households is calculated as single-parent households with dependent children under 18 years divided by total families [2]. Finally, vehicle access is measured as households without a vehicle divided by total occupied housing units [2]. These carefully defined components work together to create a comprehensive measure of social deprivation that can guide healthcare resource allocation decisions.

The SDI and SDeI can be powerful tools for identifying cancer disparities in Mississippi and for finding practical solutions to overcome them. Researchers can visualize the relationship between social deprivation and cancer outcomes by mapping SDeI scores across various geographic levels and comparing them with cancer data. Correlation studies can examine the links between SDeI scores and cancer incidence, mortality, or survival rates. At the same time, the detailed analysis of SDeI components can enlighten us about specific socioeconomic factors most strongly associated with disparities. The SDeI can also assess healthcare access by overlaying deprivation maps with CC facility locations and identifying underserved areas. Furthermore, the SDeI can guide resource allocation for cancer prevention efforts and help design targeted interventions. By using the SDeI as an evaluation metric, public health officials can track the effectiveness of their efforts and inform targeted initiatives aimed at strengthening health disparities research and reducing cancer disparities in Mississippi's most vulnerable communities [2,10].

How to "leapfrog cancer care improvement" in Mississippi?

In 2015, leaders from four preeminent cancer organizations - the American Association for Cancer Research, American Cancer Society, American Society for Clinical Oncology, and National Cancer Institute (AACRSCONCI) - initiated a collaborative effort to evaluate health disparities in the United States [10]. This joint initiative resulted in a set of strategic recommendations aimed at advancing the field of cancer health disparities research. Their objective was to provide a roadmap for public and private entities to guide targeted investments, aiming to eliminate disparities in cancer incidence, treatment quality, and patient outcomes [10]. These evidence-based recommendations seek to transform the landscape of CC by addressing longstanding inequities and improving health outcomes for all populations [10]. These recommendations can be tailored to Mississippi's unique healthcare landscape, socioeconomic factors, and population demographics, providing a framework for significant improvements in CC across the state. One of the first steps can be the determination of SDI and SDeI. In addition, new technologies and innovations (Table 3) [11-45] have to be rapidly incorporated and implemented on an ongoing basis by building an administrative infrastructure to accomplish these aims rapidly, as expanded in the five recommendations below. These efforts must be "customized" to the local needs of the state of Mississippi using SDI and SDei or their equivalent metrics that tie SDOH with CC outcomes.

**Table 3:** New technological and innovative breakthroughs in cancer care. The listed breakthroughs are only illustrative examples and not an exhaustive list.

Number	Innovation	Description (This column is modified from many sources and the sentences within “____" are reproduced verbatim. The appropriate citations follow the quotation symbols “___”.)	Immediate [within five years] usefulness index to Mississippi 1-10 (ten being most useful) *
1	CRISPR [11] A search in clinicaltrials.gov yielded 35 clinical trials [14].	This molecular biology innovation “works like a pair of scissors that can precisely delete, insert, or edit specific bits of DNA inside cells.” This “gene-editing tool emerged from a side project fueled by curiosity about how bacteria fight viruses” that led to Drs. Jennifer Doudna and Emmanuelle Charpentier winning a Nobel award in Chemistry in 2020. CRISPR-based cancer research and clinical trials are ongoing and are expected to bring far-reaching advances [12,13].	Five
2	Artificial intelligence (AI) [11]	“AI involves programming a computer to act, reason, and learn.” AI’s main benefit is it’s ability to deal with large quantities of data in analysis and pattern identifications. AI is quickly becoming an inevitable aspect of cancer research, clinical trials, prevention strategies and treatment [15,16].	Nine
3	Telehealth / telemedicine [11]	Covid 19 pandemic helped push the use of telehealth in real clinical practice. For example, the “NCI Community Oncology Research Program (NCORP) successfully incorporated or expanded telehealth practices to provide patients’ cancer treatment and care remotely” with a focus on “maximizing safety and convenience for both patients and providers across the country” in “remote health monitoring, video visits, and even in-home chemotherapy”. These interventions “make access to clinical trials and cancer care easier for more diverse groups of patients across wider geographical areas”.	Ten [The bold, italicized and underlined sentence on the left column shows the reason for the rating of ‘10’]
4	Infinium assay [11]	A “technology that reads and compares genes across people” and “used by companies like 23andMe and Ancestry, the Infinium Assay, developed by Illumina, is a process and set of tools that analyzes millions of single nucleotide polymorphisms, or SNPs, the most common type of genetic variation”. SNPs can help map genes that cause cancer and provide insight into cancer risk, progression, and development. Remarkably, “the assay was created with support from NCI’s Small Business Innovation Research program” using the US taxpayers’ monies, it is starting to be used in cancer care extensively including for example NIH’s All of Us Research Program.	Eight For example, its use in HPV-positive head & neck cancer risk assessment, the risk was associated with distinct HLA variants [18]. More importantly, some of these findings were shared with another HPV-related cancer – uterine cervical carcinoma. Similar population- based Precision Population Medicine [PPM] efforts in Mississippi can be extremely useful [19].
5	Interdisciplinary science, research and policy applications	“Scientific research that crosses traditional academic disciplines and breaks down subject silos is widely understood to be essential for the next generation of big breakthroughs and the key to solving the world’s most pressing problems” [22].	Eight [20,21,23] The problems of Mississippi can not be solved by single discipline. Only an interdisciplinary team of scientists, epidemiologists, physicians, care givers, nurses, policy makers, advocacy experts etc. can solve the cancer problem in Mississippi. This can be a ‘pilot project’ to solve Mississippi’s other health issues too.
6	Accessibility to precision (population) medicine	It is well known that Precision / Personalized Medicine [PM] or better PPM [24] that includes wider interdisciplinary disciplines is improving cancer care^[25,26]^. More important is the accessibility to PM/PPM [24,27.28].	Ten This gets a score of 10/10 since what is the benefit of a new innovation if it cannot reach out to the rural populations and the nations’ all citizens equally? [29] Also because of the potential for early diagnosis, prevention and cancer eradication potentials [30,31].
7	Liquid biopsies / MCED (multiple cancer early diagnosis) screeners	A liquid biopsy (LQB) [32] used to be defined as an intravenously collected blood sample just like sampling for cholesterol testing. This was first described in 2010 for collecting circulating tumor cells. As Alix-Panabières et al describes [32], to include the whole continuum of cancer care, the definition must be broader. Thus, LQB currently encompasses circulating cell-free tumor DNA (ctDNA), circulating cell-free RNA (miRNA and messenger RNA), extracellular vesicles, tumor-educated platelets, circulatory proteins, circulating immune cells and other immune system components in addition to circulating microbiome (liquid microbiopsy that includes circulating cell-free microbial DNA plus a defined panel of proteins and metabolites^ [32]^. Including other physiological fluids (cerebrospinal fluid, urine, bone marrow, sputum, and saliva) [32]. This definition should include MCED tests [33,34]	Eight This innovation can make ‘number 7’ (accessibility to PPM) possible, since blood samples are easier to be transported to centralized laboratories from even remote geographies. The rating of 8/10 instead of a higher rating is because this innovation is still evolving [although rapidly] and many clinical trials are ongoing.
8	Fourth industrial revolution [4IR][35,36,37]	A German engineering Professor, Klaus Schwab introduced the term in 2016 [35]. The now accepted concept is the influence of the billions of world’s population connected by “mobile devices, with unprecedented processing power, storage capacity, and access to knowledge”, combined with rapidly emerging innovations such as “artificial intelligence, robotics, the Internet of Things, 3-D printing, nanotechnology, biotechnology, materials science, and quantum computing” on many disciplines including health care and medicine [35]. For example, in cancer care, “robotic surgery, stereotaxic surgery, chemoembolization, stereotactic body radiation therapy (SBRT), cerebral navigation, monoclonal antibody drug therapy are just a few examples”. In Immunotherapy and Radiobiotherapy [39], monoclonal antibodies’ mechanisms of action are varied: “flagging cancer cells, triggering cell-membrane destruction, blocking cell growth, preventing blood vessel growth, blocking immune system inhibitors, directly attacking cancer cells, binding cancer and immune cells” [35].	Six The “merging of the physical, digital and biological worlds” [38] in 4IR is predicted to force countries, states and populations to benefit, if correct policy decision are planned, strategized and implemented [35,36,37,38]. In view of this, Mississippi has no option but to stay on the top of these developments. If Mississippi plans and executes well, we can leapfrog to a much better cancer care and outcomes. The rating of 6/10 is due to its evolving nature. However, this rating may ‘go up’ rapidly in the coming years.
9	Digital twins in health care[40,41,42]	Digital twins (DT) are “virtual replicas of real human patients, through which clinicians can gain valuable insights, optimize treatment strategies, and deliver personalized care” [40]. “Digital twins have emerged as a groundbreaking concept in personalized medicine, offering immense potential to transform health care delivery and improve patient outcomes. It is important to highlight the impact of digital twins on personalized medicine across the understanding of patient health, risk assessment, clinical trials and drug development, and patient monitoring” [42]. “Digital twins integrate data from multiple sources, including EHRs, wearable devices, genetic information, and patient-reported data. This comprehensive data integration allows health care professionals to gain a more complete picture of a patient’s health. By aggregating and analyzing diverse data sets, digital twins can identify patterns, correlations, and potential health risks that might not be evident through isolated data sources” [42].	Six DT models require enormous data collection. Fortunately, many of these data points can be collected using digital tools such ‘Wearables’. The score of 6/10 represents the rapidly evolving nature of this tool. However, its usefulness in clinical trials makes DT to have enormous potential in Mississippi. DT data can help the PPM attempts to improve citizens participation in clinical trials and personally take responsibility for their cancer care, including early diagnosis and screening schedules.
10	Wearable devices (WD) [43]	Combining many of the tools described here such as AI, telemedicine, DT and WD will provide enormous power to collect data at the ‘source; - the patient / subject’s home. With the rapid advances of the above tools, the opportunities to find solutions to cancer care problems in Mississippi with an assured improvement in outcomes and decrease in the morbidity and mortality depends on how Mississippians strategize to take advantage of these innovations.	Six The ability to make a difference in improving cancer care outcomes in Mississippi with WD, again depends on how quickly Mississippi’s leaders take actions to take advantage of these innovations and move the score to 10.

The first recommendation requires the utilization of advanced research tools and methodologies [10]. The state of Mississippi can benefit from prioritizing using sophisticated instruments to define and analyze its diverse population’s sociodemographic and economic characteristics [10]. This effort requires recruiting and retaining specially trained scientists dedicated to Mississippi's unique challenges. These experts must have state-of-the-art infrastructure and adequate funding to conduct thorough research [10]. A key focus should be collecting and analyzing the most granular data available, including detailed SDeIs and other relevant metrics [2]. By investing in these areas, Mississippi can develop a nuanced understanding of its CC landscape, enabling targeted interventions to reduce disparities in cancer incidence, treatment quality, and outcomes across the state. This data-driven approach will allow for the development of tailored strategies that address the specific needs of Mississippi's underserved communities and ultimately improve CC for all residents.

Experts also suggest the development of a specialized health disparities research network to gather a wide range of data [10]. This consortium would focus on collecting relevant biospecimens, clinical information, individual-level data, and contextual information [10,31,46-48]. This comprehensive data collection is crucial for conducting robust, hypothesis-driven health disparities research with adequate statistical power [10]. A health disparities research network would be vital in designing appropriate studies and effectively recruiting participants. Such a network would bring together interdisciplinary experts from various fields, fostering collaboration and ensuring that research efforts are well-coordinated and targeted towards addressing the most pressing health disparities issues and applying state-of-the-art precision medicine and precision population medicine concepts rapidly [10,19,24,31,39,46-48].

The third recommendation suggests that best-practice strategies must be designed and implemented to ensure that underserved patients, their healthcare providers, and relevant institutions are effectively targeted and thoroughly informed about opportunities to participate in research studies and clinical trials [10]. This approach aims to bridge the gap in representation within medical research by actively reaching out to historically underrepresented communities [10]. By developing tailored communication and engagement methods, we can increase awareness of research opportunities among these groups, potentially leading to more diverse study participation. This targeted outreach is crucial for enhancing medical research’s inclusivity and addressing health disparities through more representative scientific inquiry [10]. To achieve these goals within Mississippi, a task force focused solely on developing state-specific strategies is needed to address the state's unique underserved populations, considering factors such as income, race, geography, and education levels.

As a fourth recommendation, experts emphasized the need to cultivate a workforce of researchers well-versed in community-engaged research (CER) [10]. To support their work, academic institutions are encouraged to revise their promotion criteria to account for the extended timelines often required for CER studies [10]. Additionally, funding bodies would benefit from providing grants for CER with the understanding that the enhanced infrastructure and community outreach initiatives developed during the project should be maintained beyond the initial funding period [10]. This long-term perspective ensures that the benefits of CER extend well past the immediate research outcomes, creating lasting positive impacts on health equity. The successful implementation of these recommendations hinges on active engagement and backing from both federal and state leadership. 

As a final recommendation from experts, healthcare systems are urged to recognize their responsibility to actively monitor the care of cancer patients [10]. Given that these patients navigate a complex network of medical services, healthcare systems must implement real-time tracking mechanisms [10]. These systems should provide comprehensive insights into patient treatments and experiences. Moreover, when systemic errors or shortcomings are identified, healthcare providers have an obligation to intervene promptly. This proactive approach not only enhances patient safety but also contributes to the overall quality of CC [10]. By maintaining vigilant oversight and readiness to address issues as they arise, healthcare systems can significantly improve outcomes and experiences for patients with cancer.

The authors’ selection of the top 10 recent technological innovations is listed in Table 3, as an illustration. The implementation of AACRSCONCI’s recommendations should also be combined with rapid and efficacious ways to bring in recent advances such as those listed in Table 3 and more. Such an effort will need an interdisciplinary comprehensive approach that follows precision population medicine concepts [19,24,31,46] and can help leapfrog CC advances in the state of Mississippi, the greater Deep South of the United States, and even beyond with similar disadvantaged resource lean populations in the Global South.

This analysis of cancer disparities in Mississippi underscores the urgent need to address the complex interplay between socioeconomic factors, healthcare access, and systemic inequalities that contribute to poor health outcomes. The state's challenges, including high poverty rates and low educational attainment, mirror broader national and global trends in CC disparities. By utilizing tools such as the SDI and SDeI, we gain valuable insights into the multifaceted nature of these disparities, allowing for a more nuanced understanding of the factors influencing cancer outcomes. A multi-pronged approach is necessary to address these disparities and significantly improve CC in Mississippi. This includes investing in advanced research tools, developing specialized health disparities research networks, implementing targeted outreach strategies, fostering a workforce skilled in CER, and enhancing healthcare systems' ability to monitor and respond to cancer patients' needs in real time.

In response to the strategic recommendations, a comprehensive solution can be implemented through the establishment of the Center for Cancer Care Improvement in Mississippi (CFCCIM) perhaps in the Delta Region. The CFCCIM, as a central hub for CC improvement, would play a crucial role in coordinating and implementing the various strategies to address disparities. This initiative, jointly funded by federal and state governments along with pharmaceutical and non-governmental organizations, would set an ambitious vision to elevate state CC outcomes to national standards within a decade. The CFCCIM would be staffed by an interdisciplinary team [46], including epidemiologists, molecular biologists, disparities researchers, information communication technology (ICT) specialists [47], and community outreach professionals, ensuring a holistic approach to CC improvement [19,24,31,46,48]. Its mission would encompass CC, research, education, and clinical trials, with a specific focus on enhancing cancer screening, early detection, and implementing cutting-edge best practices.

To stay at the forefront of cancer treatment, the CFCCIM would forge center-enterprise partnerships with the industry, facilitating the rapid introduction of new technologies to Mississippi in real time. As an initial step towards realizing these concepts, the CFCCIM's first task would be to organize a stakeholders' conference in Mississippi, serving as a launchpad for this transformative initiative and bringing together key players to align efforts in improving CC across the state. By leveraging the advancements in research and technology, Mississippi has the potential to significantly improve cancer outcomes for all its residents. Moving forward, it is crucial to maintain a commitment to evidence-based strategies, community involvement, and ongoing progress monitoring to ensure that advancements in CC reach all populations equitably. Further research and long-term studies will be essential to evaluate the effectiveness of these interventions and to continuously refine our approach to reducing CC disparities in Mississippi and beyond.

## Conclusions

This review and perspective bring an interdisciplinary approach to an environment of rapidly changing digital and biological breakthroughs and innovations in CC. On the one hand, the global burden of cancer is increasing, especially among underserved communities - in the first world nations, as well as in the Global South. On the other hand, there is dynamic scientific progress that promises potential rapid solutions to solve the burgeoning cancer problem. To make these potentials a reality, a team science and interdisciplinary collaborative approach is the only option. A successful demonstrative, comprehensive, interdisciplinary, strategic, and forward-looking project to solve Mississippi's CC issues will serve geographies and communities far beyond the state of Mississippi.
